# Near-infrared spectroscopy for early selection of waxy cassava clones via seed analysis

**DOI:** 10.3389/fpls.2023.1089759

**Published:** 2023-01-23

**Authors:** Massaine Bandeira e Sousa, Juraci Souza Sampaio Filho, Luciano Rogerio Braatz de Andrade, Eder Jorge de Oliveira

**Affiliations:** ^1^ Embrapa Mandioca e Fruticultura, Cruz das Almas, Bahia, Brazil; ^2^ Universidade Federal do Recôncavo da Bahia, Cruz das Almas, Bahia, Brazil

**Keywords:** amylopectin, amylose, classification models, Manihot esculenta Crantz, portable NIRS

## Abstract

Cassava (*Manihot esculenta* Crantz) starch consists of amylopectin and amylose, with its properties determined by the proportion of these two polymers. Waxy starches contain at least 95% amylopectin. In the food industry, waxy starches are advantageous, with pastes that are more stable towards retrogradation, while high-amylose starches are used as resistant starches. This study aimed to associate near-infrared spectrophotometry (NIRS) spectra with the waxy phenotype in cassava seeds and develop an accurate classification model for indirect selection of plants. A total of 1127 F_2_ seeds were obtained from controlled crosses performed between 77 F_1_ genotypes (wild-type, *Wx*_). Seeds were individually identified, and spectral data were obtained *via* NIRS using a benchtop NIRFlex N-500 and a portable SCiO device spectrometer. Four classification models were assessed for waxy cassava genotype identification: k-nearest neighbor algorithm (KNN), C5.0 decision tree (CDT), parallel random forest (parRF), and eXtreme Gradient Boosting (XGB). Spectral data were divided between a training set (80%) and a testing set (20%). The accuracy, based on NIRFlex N-500 spectral data, ranged from 0.86 (parRF) to 0.92 (XGB). The Kappa index displayed a similar trend as the accuracy, considering the lowest value for the parRF method (0.39) and the highest value for XGB (0.71). For the SCiO device, the accuracy (0.88−0.89) was similar among the four models evaluated. However, the Kappa index was lower than that of the NIRFlex N-500, and this index ranged from 0 (parRF) to 0.16 (KNN and CDT). Therefore, despite the high accuracy these last models are incapable of correctly classifying waxy and non-waxy clones based on the SCiO device spectra. A confusion matrix was performed to demonstrate the classification model results in the testing set. For both NIRS, the models were efficient in classifying non-waxy clones, with values ranging from 96−100%. However, the NIRS differed in the potential to predict waxy genotype class. For the NIRFlex N-500, the percentage ranged from 30% (parRF) to 70% (XGB). In general, the models tended to classify waxy genotypes as non-waxy, mainly SCiO. Therefore, the use of NIRS can perform early selection of cassava seeds with a waxy phenotype.

## 1 Introduction

Cassava (*Manihot esculenta* Crantz) is one of the most accessible and consumed sources of carbohydrates, being widely used as processed products and in its natural form as animal and human food. In Brazil, cassava has recently increased its value due to its different available applications, especially in the food industry. Starch is the main storage carbohydrate in plants, with its biosynthesis occurring in seeds, tubers, fruits, roots, and leaves. It is essential not only in the life cycle of plants but also in human nutrition as it provides large amounts of energy (Li et al., 2019). Along with corn, potato, wheat, and rice, cassava is one of the main commercial sources of starch globally (Agama-Acevedo et al., 2019).

Cassava starch comprises two types of glucose polymers, amylose and amylopectin, whose composition ranges from 15−27% amylose, with an average of 21% (Sánchez et al., 2009; Santos et al., 2021). Waxy starch comprises at least 95% amylopectin, and this is associated with certain advantages, including less starch retrogradation and syneresis from starch pastes during freeze/thaw cycles; this prevents the reduction of sensory quality and shelf life of processed foods (Demiate and Kotovicz, 2011; Wang et al., 2015; Morante et al., 2016). The waxy starches of roots and tubers, such as cassava and potato, compared to cereal waxy starches provide clearer gels, with a mild or neutral flavor (Koehorst-van Putten et al., 2012), and different, higher viscosity gel textures (Sánchez et al., 2010). Additionally, they are used in food products, such as nuggets, to provide crunchiness and prevent excessive oil penetration during preparation, and in the gummies industry, they provide 25−50% of the total starch used in the formulations (Cai et al., 2010; Li et al., 2019).

Developing cassava varieties with waxy starch has become an important goal for cassava breeders. However, the recessive nature of the trait and the long reproductive cycle of cassava make the selection of waxy genotypes relatively complex. The introgression of recessive traits requires multiple generations of recombination to reduce the linkage drag of unwanted alleles of the parental genotype that contain the waxy mutation(s), such as low dry matter content and root yield (Karlström et al., 2016). A crossing between an elite non-waxy and a waxy variety, which contains many undesirable genes besides the starch mutation, is expected to have a 100% frequency of non-waxy genotypes (wild-type, *Wx*_) in the F_1_ generation and segregation of 3:1 (non-waxy:waxy) in the F_2_ generation. Nonetheless, due to the high heterozygosity present in the population and the little variability between the waxy starch sources, the selected genotypes have lower or similar yield potential and lower starch content than the parental genotypes (Karlström et al., 2016; Rojanaridpiched et al., 2020; Ceballos et al., 2021). This result is a consequence of inbreeding depression caused by the increased frequency of homozygous genes, often deleterious, whose expressions are repressed in their heterozygous form. Currently, there are efforts to increase recombination cycles to maintain the waxy gene in homozygosity and break undesirable genetic linkage or even increase heterozygosity for loci associated with important agronomic attributes in cassava.

Genomic studies have enabled the identification of target genes that control amylose and amylopectin synthesis and enabled the selection of markers associated with these genes with potential use in marker-assisted selection (MAS) (Aiemnaka et al., 2012; Carmo et al., 2020). Starch biosynthesis is genetically controlled by target genes, including granule-bound starch synthases (GBSS), soluble starch synthases (SSS), starch branching enzyme (SBE or BE), debranching enzyme (DBE), and protein targeting to starch (PTST) (Zeeman et al., 2010; Bahaji et al., 2014; Seung et al., 2015; Seung et al., 2017). The SSS, BE, and DBE genes are involved in amylopectin synthesis, and GBSS and PTST are enzymes related to amylose biosynthesis in plants, including cassava (Zhao et al., 2011; Bull et al., 2018).

GBSSI-related SNP markers have not proven useful for MAS in populations with different genetic backgrounds (Aiemnaka et al., 2012; Carmo et al., 2020). Alternatively, the phenotypic identification between waxy and non-waxy genotypes is usually determined by staining the roots with iodine, which is a chemical method. Non-waxy, starchy roots stain dark blue due to the presence of amylose, and waxy phenotypes stain reddish brown (Ceballos et al., 2007). However, the screening of waxy clones by the iodine method requires the presence of tuberous roots, and for this reason, in most genetic breeding programs, the selection is conducted during or close to harvest, 10 months after planting. Thus, an evident disadvantage of this process is the difficulty of the early selection of waxy clones. Therefore, the development of rapid methodologies to identify the waxy phenotype, regardless of the genetic origin of the mutation, can help optimize the selection process.

Near-infrared spectroscopy (NIRS) technologies have been used with great accuracy as auxiliary tools in the phenotyping process, aiming to accelerate the selection steps. The performance of NIRS is comparable to other analytical chemistry methods with advantages including shorter analysis time, early evaluation, bulk sample analysis per day, and non-destruction of samples (Ikeogu et al., 2017). Near-infrared (NIR) electromagnetic region radiation (700−2500 nm) is absorbed by water and organic compounds, including carbohydrates, proteins, lipids, or alcohols (Agelet and Hurburgh, 2014). Therefore, NIRS can serve as an important predictor of these compounds in organic substances.

Carmo et al. (2019) evaluated Fourier-transform near-infrared spectroscopy (FT-NIRS) for indirect, early identification of waxy starch cassava genotypes by screening samples of dried, macerated leaves. In this study, the distribution between the classes of waxy and non-waxy genotypes was similar, and the results showed high accuracy, deeming it a potential technique for the classification of waxy genotypes. However, despite this analysis being earlier than the analysis of iodine in tuberous roots, it is still necessary to germinate a large batch of seeds in the greenhouse, collect and identify leaf samples, dry macerate, and perform screening *via* NIRS. Considering the typical segregation of genes with recessive inheritance, only 25% of the F_2_ seeds will be classified as waxy and, therefore, most of the investments in germination and sampling for evaluation *via* NIRS were conducted in unwanted samples. Thus, the development of waxy and non-waxy seed classification models allows for an early, non-destructive seed selection that saves time and resources, ensuring only waxy seeds followed in the selection pipeline.

In fact, NIRS has been used as an efficient tool for classifying and predicting seed germination capacity, quality, and vigor (Al-Amery et al., 2018; Medeiros et al., 2020; Mortensen et al., 2021). This approach allows for the selection and classification of seeds according to specific traits without damaging or changing seed properties. Analyses in the endosperm of waxy, normal, and sweet corn varieties have demonstrated the ability to detect differences between amylopectin and amylose structures, shape, and size of starch granules as starch is synthesized within amyloplasts (Yu et al., 2015). This is useful for the selection of plants of interest in breeding programs.

With the interest in early selection of waxy genotypes, this study aimed to associate near-infrared spectrophotometry spectra with the waxy phenotype in cassava seeds and develop an accurate classification model for indirect selection of plants soon after the performance of the crossing’s blocks.

## 2 Material and methods

### 2.1 Obtaining seeds and collecting spectra using NIRS

Two generations of recombination were performed to obtain segregating populations for the waxy gene. The genotypes were cultivated in a two-crossing blocks field located in the experimental area of Embrapa Cassava and Fruits in Cruz das Almas, Bahia, Brazil (12°39′25″ S, 39°07′27″W, 226 m altitude). The parent plants of the F_1_ and F_2_ populations were planted from 2016−2017 and 2018−2019, respectively. The weather conditions are hot, humid, and tropical (Aw/Am, according to the Koppen classification) with a photoperiod throughout the year of approximately 12 hours (Souza et al., 2020). Cuttings (16–20 cm long) with 5–7 buds were grown under rainfed conditions in plots containing two rows with eight plants each, spaced 1.20 m between rows and 0.80 m between plants. All cultivation practices were adopted by Souza et al. (2016).

The F_1_ population was achieved through crossing a waxy (*wxwx*) genotype (Cassava-7909) with three non-waxy (wild-type, *Wx* _) genotypes (BGM-0131, BGM-0728, and BGM-0935). For the F_2_ population, controlled crosses were randomly performed among 77 F_1_ genotypes (wild-type, *Wx*_) to produce F_2_ seeds. These parents were generated through crosses from three different F_1_ families, with 13, 35, and 28 genotypes each. Overall, 39 genotypes were used as both male and female parents, while 69 and 46 were used only as female or male parents, respectively. In total, 197 F_2_ families and 1127 F_2_ seeds were obtained.

To prevent insect pollination, the female flowers were protected by a voile-type fabric bag 24 hours before anthesis, which is easily identifiable by experienced field workers. Male flowers, immediately following anthesis, were collected from 7–9 a.m., and the crosses were performed between 9 a.m. and 4 p.m. by distributing pollen grains on stigmas. One male flower was used to pollinate up to three female flowers, depending on the amount of pollen available. The female flowers were protected again, as previously described, shortly after pollination. One cross was defined as a single pollination event. After identifying female flowers ready for pollination, crosses were performed in one to four flowers per inflorescence, and the remaining flowers were removed. The protection bag covered the inflorescence until the seeds were released and collected, which occurred approximately 2−3 months post pollination. Each seed was labelled with the family information and the seed number, and they were individually stored in plastic bags in a refrigerator (10 ± 2°C) until further analysis.

Seed spectra were obtained in a laboratory at a room temperature of 22°C through ultraviolet-visible and near-infrared spectrophotometry using a benchtop NIRFlex N-500 spectrometer (Büchi, Flawil, Switzerland) and a portable SCiO (Consumer Physics, Tel-Aviv, Israel). The spectra were obtained by placing the samples (one whole seed at a time), directly at the output of the infrared source of the device. Four measurements were taken per seed using the NIRFlex N-500, with a wavelength ranging from 800–2500 nm (12500–4000 cm^−1^). The NIRFlex N-500 was operated in diffuse reflectance mode at a spectral resolution of 8 cm^-1^, interpolated at 4 cm^-1^, resulting in 1501 data points per spectrum. For the SCiO portable device, three measurements were collected per seed (N=334) in diffuse reflectance mode with wavelengths ranging from 740–1070 nm (13.514–9.346 cm^−1^). This device has a set of 12 photodiode detectors, each with a separate optical filter. The average spectral resolution of SCiO was 13 cm^−1^, with the lowest resolution (18 cm^−1^) found in the highest wavenumbers and the highest resolution (9 cm^−1^) in the lowest wavenumbers. The SCiO™ Lab online app (Consumer Physics Inc., Tel-Aviv, Israel) was used for data collection, storage, and analysis.

### 2.2 Seedling trial and phenotypic data collection

After collecting the spectral data, the 1127 F_2_ seeds were sown in 290 cm^3^ plastic tubes and placed in trays in a greenhouse at 32 ± 3°C. The tube substrate comprised vermiculite and washed sand (1:1 ratio) in the upper quarter, and the lower three quarters was composed of vermiculite, sand, and coconut fiber (ratio 1:2:1) as well as 15 mg each of single superphosphate and ammonium sulfate. The seedlings were transplanted to the field when approximately 30 cm in height, around 45 days after germination. The cultural treatments were performed according to Souza et al. (2016).

The harvest was conducted at 10 months of age, and the evaluation was performed using the 2% iodine staining test (2 g Kl and 0.2 g I^2^ in distilled water); stain was applied to the cross section of at least three roots of the seedlings for the identification of the type of starch (Karlström et al., 2016; Morante et al., 2016). A dark blue color in the treated root indicated the presence of amylose (non-waxy genotype), and a reddish-brown color indicated no or low amylose content (waxy genotype) (Denyer et al., 2001).

### 2.3 Discriminant analysis of principal components

The population structure of the genotypes was determined by principal component discriminant analysis (DAPC) (Ivandic et al., 2002), using the adegenet package (Jombart, 2008) of the R software version 4.1.3 (R Core Team, 2021). The find.clusters() function was used in detecting the number of clusters in the population. The function uses K-means clustering, which deconstructs the total variation of a variable into components between groups and within the group. The best number of subpopulations was chosen by the smallest Bayesian Information Criterion (BIC). The groups were plotted on a scatterplot of the first and second linear discriminant of the DAPC.

### 2.4 Pre-processing and adjustment of classification models

Several pre-processing techniques were evaluated to ensure spectral data reliability such as: first-order derivative (1st); detrend (DT); multiplicative scatter correction (MSC) and standard normal variation (SNV); Combined pretreatment methods, first-order derivative-detrend (1st-DT); first-order derivative-multiplicative scatter correction (1st-MSC); detrend-multiplicative scatter correction (DT-MSC); and first-order derivative with Savitzky–Golay-detrend (1st-SG-DT). The first-order derivative was used to substracted the influence of background and baseline drift, DT was used to eliminate the baseline drift in the spectra, and MSC and SNV methods were used to eliminate the scattering multiplicative interferences in the spectral signal.

The spectra were pre-processed for above tecniques and then smoothed with an N=11 filter at each end of the spectral set for noise reduction (Savitzky and Golay, 1964). The DT, MSC, SNV, and SG were implemented by the functions detrend(), msc(), standardNormalVariate(), and savitzkyGolay(), respectively, from the prospectr package (Stevens and Ramirez-Lopez, 2022) implemented in the R software version 4.1.3.

After pre-processing, the spectral data were arranged in an X matrix (predictors), and the starch type data (waxy and non-waxy) were allocated in a Y vector (response). Four classification models were assessed for waxy cassava genotype identification: k-nearest neighbor algorithm (KNN) (Cover and Hart, 1967), C5.0 decision tree (CDT) (Freund and Schapire, 1997), parallel random forest (parRF) (Breiman, 2001), and eXtreme Gradient Boosting (XGB) (Chen and Guestrin, 2016).

KNN is a commonly used non-parametric algorithm in Machine Learning. It is mathematically simple and based on the determination of distances, often Euclidean, between an unknown object and each of the objects in the training set. Thus, the smallest distance is selected for assigning the members of a given class. With k representing the number of neighbors, the k-nearest objects of the unknown sample are selected, and a majority rule is applied: the unknown sample is classified in the class to which most k objects belong. The choice of k is optimized by calculating the predictive power with different values of k.

C5.0 is an algorithm based on decision trees (Elsayad et al., 2020), which involve a set of decision nodes, among which the root and each internal node are labeled with a question (Pradhan, 2013). The arcs descend from each root node to leaf nodes, where a solution to the associated issue is offered. A split is created at each node by taking a binary decision, which separates a class or multiple classes from the global dataset.

The RF algorithm is a type of ensemble learning and is a method that generates several decision trees and combines the result of the classification from each of them. This combination of models makes it more powerful than Decision Tree. The algorithm works by growing a set of regression trees based on binary recursive partitioning, where the algorithm begins with a number of bootstrap samples from the predictor space (original data) (Cutler et al., 2012).

XGBoost is a machine learning algorithm based on a gradient boosting decision tree (GDBT) (Chen and Guestrin, 2016). XGBoost is an extension of RF (Svetnik et al., 2003), and, as a differential, it can use a regularization term to further reduce overfitting, improve prediction accuracy, and decrease the time needed to build decision trees (Luckner et al., 2017). All data analyses were performed with the R software version 4.1.3 using the caret package (Kuhn, 2008).

The selection of wavelengths with relative importance was conducted using the XGB model, as it automatically provides estimates of the importance of the variables. Variables with relative importance (≥30%) were selected. For this, the varImp() function from the caret package of the R software version 4.1.3 was used, which automatically scales importance scores between 0 and 100.

### 2.5 Cross-validation and external validation

Data were divided into a training set, for model development purpose, (80% of the data) and a testing set used as independent samples to test the classification models (used to obtain the confusion matrix), both with equitable distribution of genotypes according to the type of starch. The model performances were evaluated in the training set based on cross-validation, consisting of 10 repetitions with 5-folds each. Parameters that provide the best fit to the data were selected for each model evaluated (**Table 1**). The overall effectiveness of the classification models was assessed based on mean values of accuracy and Cohen’s Kappa statistic (unweighted) (Cohen, 1960), obtained in each repetition of the cross-validation. The accuracy was determined using the equation 1:

**Table 1:** Parameters used in the k-nearest neighbor algorithm (KNN), C5.0 decision tree (CDT), eXtreme Gradient Boosting (XGB), and parallel random forest (parRF) classification models using all variables and selected variables with relative importance (≥30%) using the XGB model.

Models	Parameters	NIRFlex N-500		SCiO	
		All variables	Selected variables	All variables	Selected variables
KNN	K	5	5	7	7
CDT	trials, model, and winnow	20, tree, and TRUE	20, tree, and FALSE	20, tree, and FALSE	20, tree, and FALSE
XGB	nrounds, lambda, alpha, and eta	150, 1e^-4^, 0, and 0.3	150, 1e^-4^, 0.1, and 0.3	50, 0, 0, and 0.3	50, 0.1, 0.1, and 0.3
ParRF	mtry*	1459	27	2	2

*number of predictors.


(1) $$Accuracy=\frac{tp+tn}{tp+fn+fp+tn}$$

where *tp* corresponds to the number of correctly recognized class examples (true positives), *tn* is the number of correctly recognized examples that do not belong to the class (true negatives), *fp* are examples that were incorrectly assigned to the class (false positives), and *fn* are examples that were not recognized as class examples (false negatives). The Kappa index is based on the number of concordant responses defined by equation 2:


(2) $$Kappa=\frac{p_{o}+p_{e}}{1-p_{e}}$$

where *p_o_* is the proportion of units that agreed, and *p_e_* is the proportion of units for which agreement is expected by chance. This index indicates how well the models can correctly classify the two analyzed classes, and the closer to one, the greater the detection power.

The testing set (20% of the data) consisted of 225 and 67 genotypes for NIRFlex N-500 and SCiO, respectively. The prediction performance was evaluated with parameters generated from a confusion matrix. The parameters were accuracy, Kappa index, sensitivity, and specificity. Sensitivity measures the probability of the classifier hitting true positives $\left(\frac{tp}{tp+fn}\right)$ , while specificity measures the probability of hitting true negatives $\left(\frac{tn}{tn+fn}\right)$ .

## 3 Results

### 3.1 Segregation and clustering of clones *via* multivariate analysis

Among the 1127 seedlings, 21.3% had waxy starch genotypes. Of the 197 families, 85 were used to assess the frequency of segregation for the mutant phenotype (Waxy – *wxwx*) because they had four or more individuals per family. As the population originated from the cross between waxy parents (*wxwx*) with a known genotype and non-waxy parents (wild-type, *Wx*_) with unknown genotypes, the expected frequencies of 3:1 and 1:1 were considered for the two possibilities of the non-waxy parent. As expected, the observed distribution of phenotypic classes in 86% of the evaluated families adjusted to a single-gene Mendelian inheritance (flex **Table S1**).

**Figure 1:**
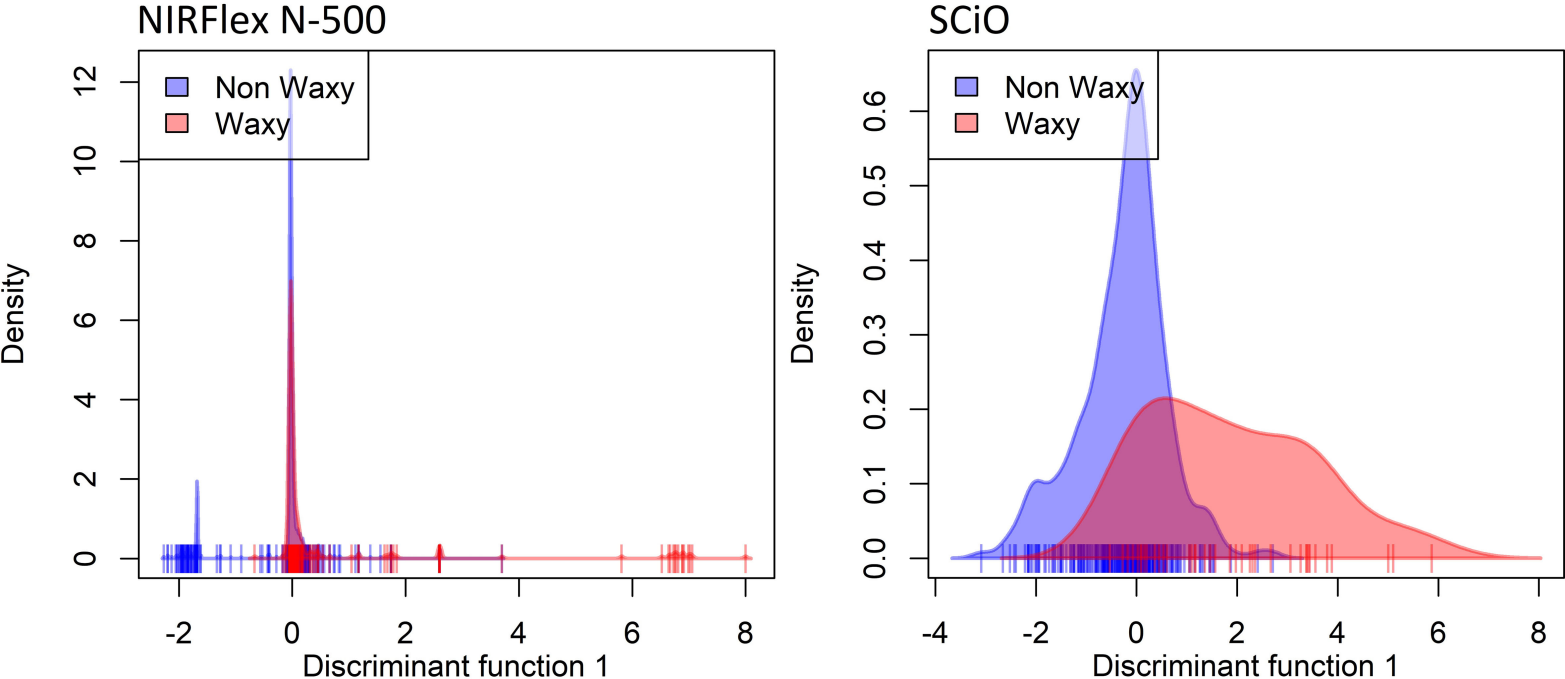
Density plot on the first discriminant function showing discriminant analysis of principal components (DAPC) based on near-infrared (NIR) spectral data obtained by NIRFlex N-500 and SCiO equipment, considering contrasting cassava genotypes for waxy and non-waxy starches.

Both the spectral data collected by the NIRFlex N-500 (240 waxy and 887 non-waxy clones) and the SCiO portable NIR (291 waxy and 44 non-waxy clones) were used to assess the potential for classifying cassava genotypes based on a waxy phenotype. The density distributions of the waxy and non-waxy clones, were determined for each NIR equipment (**Figure 1**). It can be observed from the density curves that both equipments displayed overlapping curves, which represent areas of confusion, with the diferentiation between the groups not being clear by visual analysis.

### 3.2 Development of classification models

To evaluate the efficiency of the pre-processing techiniques were used the parameters Accuracy and the Kappa index from the KNN classification method (**Figure S1**). In general, according to cross-validation the results were similar between the pre-processing techniques, with lower performance when using the raw data without pre-processing. The 1st and MSC combination was selected to proceed with the analyses.

**Figure 2:**
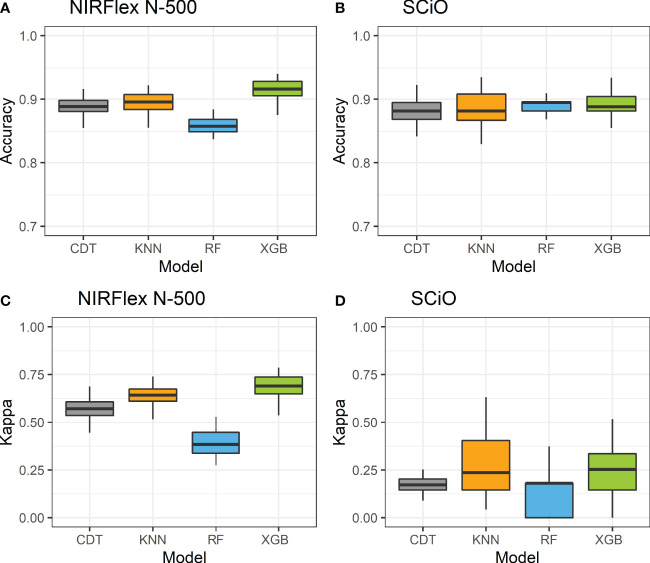
Accuracy **(A, C)** and kappa index **(B, D)** of cross-validation of classification models based on NIRFlex N-500 and SCiO near-infrared spectra evaluated in cassava seeds contrasting for waxy and non-waxy starch. KNN, k-nearest neighbor algorithm; CDT, C5.0 decision tree; XGB, eXtreme Gradient Boosting; parRF, parallel random forest.

Accuracy and the Kappa index were used as parameters to evaluate the efficiency of the models with the best fit in the classification of waxy and non-waxy clones. Generally, the classification accuracy using NIRFlex N-500 spectral data varied among the different models analyzed. According to cross-validation, the accuracy ranged from 0.86 (parRF) to 0.92 (XGB) (**Figure 2**; **Table 2**). The Kappa index displayed a similar trend as the accuracy, considering the lowest value for the parRF method (0.39) and the highest value for XGB (0.69). Regarding the NIRFlex N-500 spectra collected, although the KNN classification method has presented similar accuracy (0.90) to the XGB model, the Kappa index was considerably lower (0.64) than the XGB.

**Table 2:** Cross-validation parameters of the k-nearest neighbor algorithm (KNN), eXtreme Gradient Boosting (XGB), C5.0 decision tree (CDT) and parallel random forest (parRF) classification models obtained through spectral data analysis from the NIRFlex N-500 and SCiO in cassava seeds with waxy and non-waxy starch.

Models*		NIRFlex N-500		SCiO	
		Accuracy	Kappa	Accuracy	Kappa
All spectra	KNN	0.90 ± 0.01	0.64 ± 0.05	0.88 ± 0.02	0.22 ± 0.14
	CDT	0.89 ± 0.02	0.57 ± 0.08	0.88 ± 0.02	0.05 ± 0.15
	XGB	0.92 ± 0.01	0.69 ± 0.05	0.89 ± 0.02	0.20 ± 0.12
	parRF	0.86 ± 0.01	0.39 ± 0.06	0.89 ± 0.01	0.13 ± 0.10
Selected spectra	KNN_Sel	0.89 ± 0.02	0.61 ± 0.06	0.89 ± 0.02	0.26 ± 0.14
	CDT_Sel	0.92 ± 0.01	0.73 ± 0.06	0.89 ± 0.02	0.23 ± 0.17
	XGB_Sel	0.95 ± 0.01	0.82 ± 0.04	0.90 ± 0.02	0.37 ± 0.16
	parRF_Sel	0.92 ± 0.01	0.72 ± 0.05	0.89 ± 0.01	0.14 ± 0.13

* Sel: models using variables selected according to their relative importance by the xgbLinear model.

Regarding NIRS SCiO, the classification accuracy was similar among the four models evaluated, with values ranging between 0.87 (CDT) and 0.89 (parRF and XGB). However, the Kappa index was lower than that of the NIRFlex N-500, and this index ranged from 0.05 (CDT) to 0.22 (KNN). These results show that, despite high accuracy values, these models, especially CDT, are incapable of correctly classifying waxy and non-waxy clones based on the SCiO device spectra.

**Figure 3:**
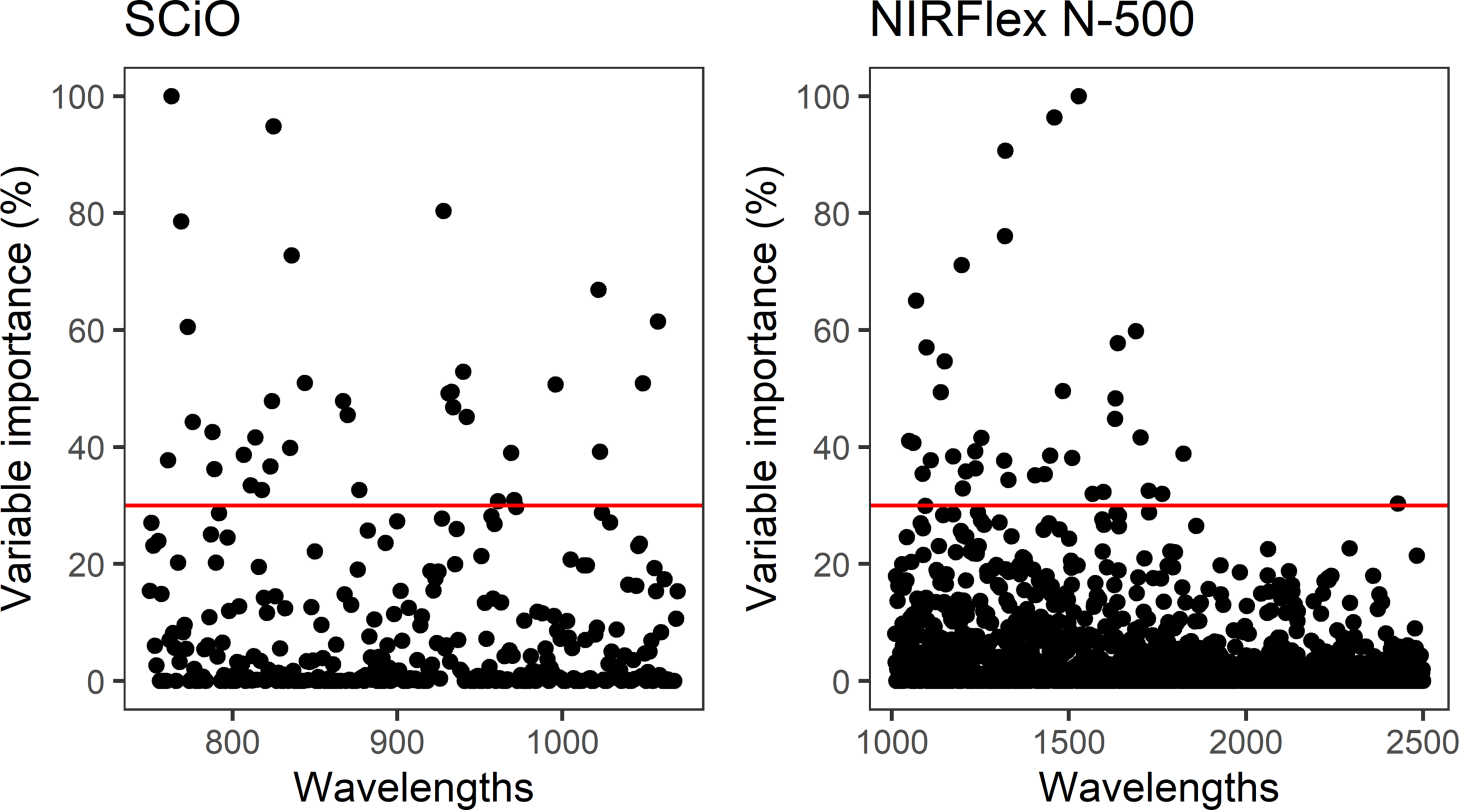
Relative importance of wavelengths collected by NIRFlex N-500 and SCiO equipment for classification of the waxy phenotype in cassava based on the eXtreme Gradient Boosting classification model.

Despite high accuracy in classifying the waxy phenotype early during the seed stage, especially in the NIRFlex N-500 spectra, the possibility of improving classification accuracy was investigated further considering the selection of variables according to the importance scores of the spectra based on the XGB model. This was warranted because spectroscopic techniques tend to generate a high number of variables (wavelengths) with noise which are highly correlated, which reinforces the importance of removing non-informative variables. Thus, the construction of consistent classification and prediction models is possible, reducing the risk of inferences and the computational cost of the analyses. Thirty seven and 34 wavelengths were selected for the NIRFlex N-500 and the SCiO, respectively, with relative importance (≥30%) (**Figure 3**).

**Figure 4:**
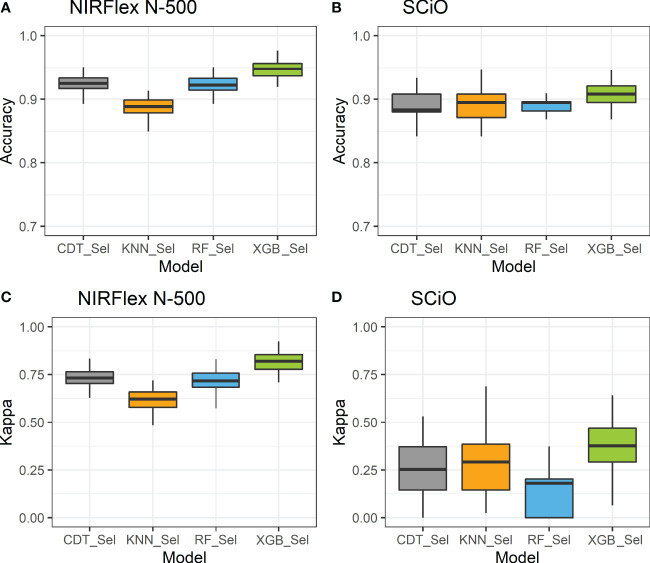
Accuracy **(A, B)** and Kappa index **(C, D)** of cross-validation of classification models based on NIRFlex N-500 and SCiO near-infrared spectra evaluated in cassava seeds contrasting for waxy and non-waxy starch. KNN, k-nearest neighbor algorithm; CDT, C5.0 decision tree; XGB, eXtreme Gradient Boosting; parRF, parallel random forest; Sel, models using variables selected according to relative importance by the XGB model.

Overall, for the NIRFlex N-500, models built on the most important spectra only resulted in an increase in classification accuracy and Kappa index estimates compared to models built on all spectra, excluding the KNN model. The CDT and XGB models resulted in an average increase of 3.7% in accuracy, while the parRF model showed a 7% increase. Furthermore, the Kappa index significantly increased from 0.57, 0.69, and 0.39 to 0.73, 0.82, and 0.72 for the CDT, XGB and parRF models, respectively (**Figure 4**; **Table 2**). However, in relation to SCiO, the accuracy estimates remained practically unchanged after the selection of the most important spectra. Alternatively, the Kappa index increased significantly from 0.05 to 0.23 (CDT), and from 0.20 to 0.37 (XGB) (**Figure 4**). However, Kappa index estimates are considered very low (< 0.37) and highly biased in their estimates (**Table 2**).

### Predictive capacity of classification models

**Figure 5:**
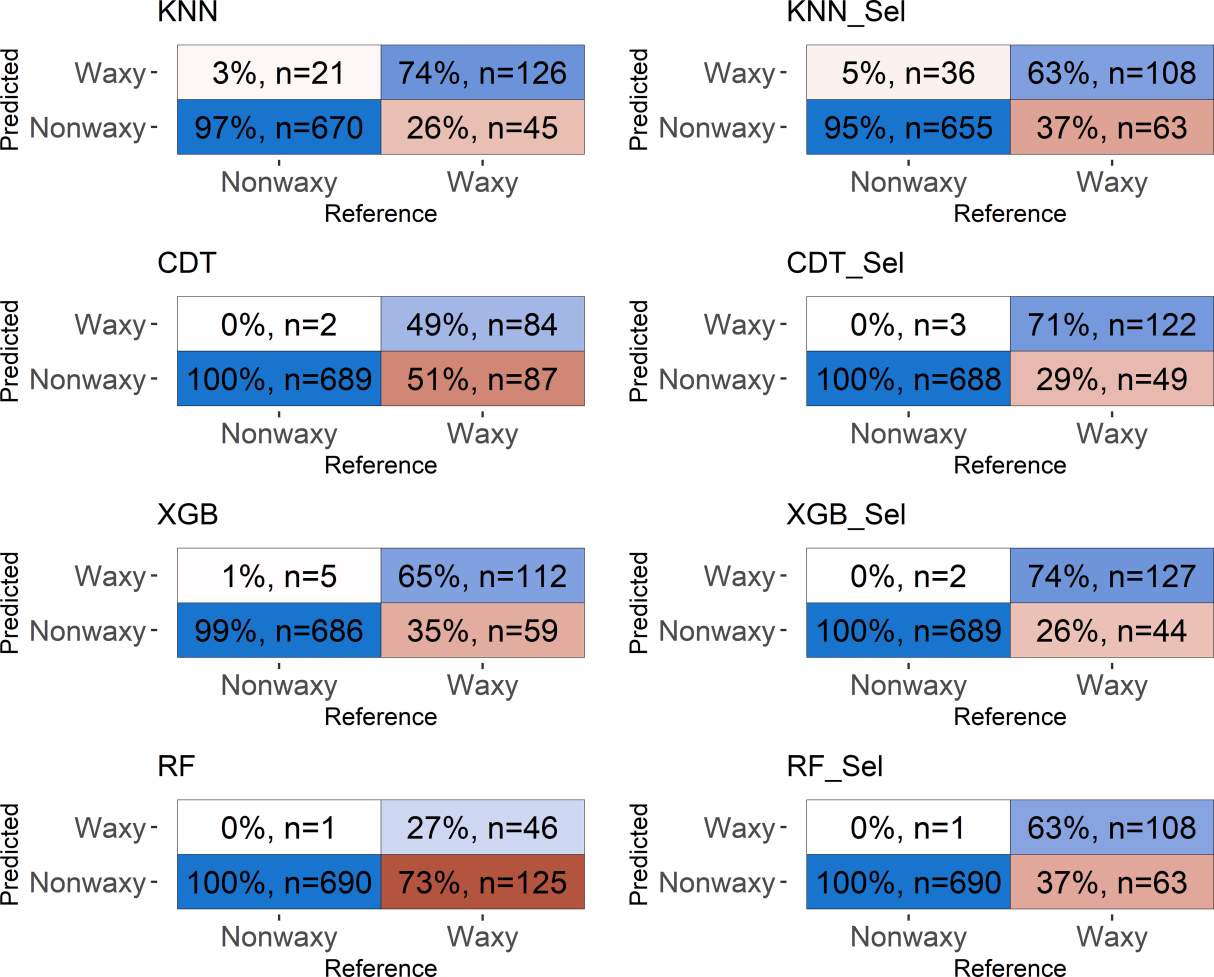
Confusion matrix of the testing set considering classification models based on near-infrared spectra by NIRFlex N-500 evaluated in cassava seeds contrasting for waxy and non-waxy starch. KNN, k-nearest neighbor algorithm; CDT, C5.0 decision tree; XGB, eXtreme Gradient Boosting; parRF, parallel random forest; Sel, models using variables selected according to relative importance by the XGB model.

**Figure 6:**
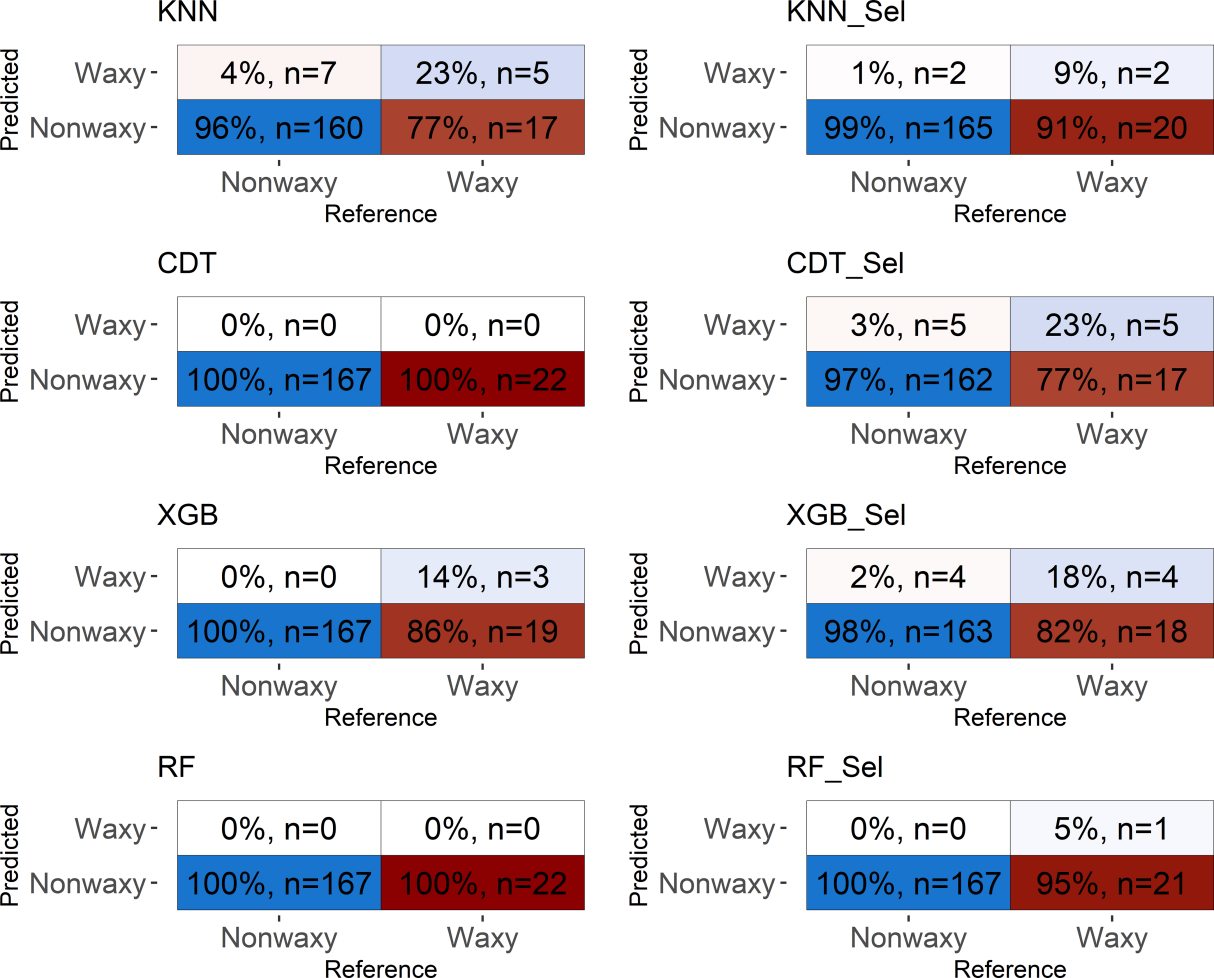
Confusion matrix performed in the testing set considering classification models based on near-infrared spectra by SCiO evaluated in cassava seeds contrasting for waxy and non-waxy starch. KNN, k-nearest neighbor algorithm; CDT, C5.0 decision tree; XGB, eXtreme Gradient Boosting; parRF, parallel random forest; Sel, models using variables selected according to relative importance by the XGB model.

The predictive capacity of the models was evaluated based on the accuracy, Kappa index, sensitivity, and specificity generated from the confusion matrix obtained by predicting the models in the testing set (**Table 3**; **Figures 5**, **6**). Considering the testing population, high classification accuracy was identified for both NIRSs. The accuracies ranged from 0.85 (parRF) to 0.95 (XGB _Sel) for the NIRFlex N-500 (**Table 3**). The Kappa index was high (>0.60), except for the parRF model with a value of 0.37 (**Table 3**). Like cross-validation, the selection of the most important spectra for model calibration provided an increase in the accuracy values and, more importantly, in the Kappa index, excluding the KNN model.

**Table 3:** Parameters from confusion matrix associated with grading efficiency of contrasting cassava seeds for waxy and non-waxy starch based on near-infrared (NIR) spectra collected by NIRFlex N-500 and SCiO equipment in test samples.

Models*		NIRFlex N-500				SCiO			
		Accuracy	Kappa	Sensitivity	Specificity	Accuracy	Kappa	Sensitivity	Specificity
All spectra	KNN	0.92	0.74	0.97	0.73	0.87	0.23	0.96	0.23
	CDT	0.90	0.60	1.00	0.49	0.89	0.19	0.99	0.14
	XGB	0.93	0.74	0.99	0.65	0.90	0.22	1.00	0.14
	parRF	0.85	0.37	1.00	0.27	0.88	0	1.00	0
Selected spectra	KNN	0.89	0.62	0.95	0.63	0.88	0.12	0.99	0.09
	CDT	0.94	0.79	1.00	0.71	0.88	0.26	0.97	0.23
	XGB	0.95	0.82	1.00	0.74	0.88	0.22	0.98	0.18
	parRF	0.93	0.73	1.00	0.63	0.89	0.08	1.00	0.05

* KNN, k-nearest neighbor algorithm; CDT, C5.0 decision tree; XGB, eXtreme Gradient Boosting; parRF, parallel random forest; Sel, models using variables selected according to their relative importance by the XGB model.

Confusion matrix based on the spectra collected by SCiO resulted in similar values of accuracy and Kappa indices, regardless of whether the model uses all spectra or only the most important for the classification of waxy clones. Again, although the SCiO spectra resulted in high classification accuracies, the capability of reliable detection among the analyzed classes was null.

Overall, values equal to or close to one were obtained for sensitivity, indicating that the models were able to predict the true positives of each class. Specificity values ranged between 0.27−0.74, for NIRFlex N-500, and were close to zero for SCiO (**Table 3**). This result indicates that most models were not efficient in predicting the true negatives of the evaluated classes. The two classes evaluated present an imbalance in relation to the number of clones that comprise each class. Therefore, the differences in sensitivity and specificity estimates are attributed to this imbalance between the classes since the confusion matrix considers the non-waxy class as positive and waxy as negative.

The confusion matrix displays the results of classifying the different models in the external validation set (**Figures 5**, **6**). For both NIRSs, the models were efficient in classifying non-waxy clones (considered the “positive” class) with hit percentages ranging between 95−100%. However, the NIRSs differ in the prediction potential of the waxy clone class. For the NIRFlex N-500, the hit percentage ranged from 27% (parRF) to 74% (KNN and XGB_Sel). In general, the models tended to classify waxy genotypes as non-waxy, especially for SCiO equipment.

## 4 Discussion

### 4.1 Evaluation of waxy phenotype classification efficiency

Several studies employ molecular markers to understand the genetic control of the waxy genotype, which guides the crossing planning of accessions, since the waxy phenotype is expressed in the recessive condition (Aiemnaka et al., 2012; Carmo et al., 2020). However, despite the development of protocols that allow the use of selection assisted by molecular markers related to the GBSSI (granule-bound starch synthase I) gene derived from the waxy starch source AM206-5, there remain obstacles when the population has a different genetic origin than the AM206-5 source (Carmo et al., 2020). Therefore, using technologies that allow a faster, earlier selection of waxy genotypes is desirable in the most diverse breeding programs.

In the present study, seeds from segregating populations of cassava for waxy starch were used as sample material for the identification/classification of waxy and non-waxy genotypes by near-infrared spectroscopy (NIRS). A previous study using spectral data collected from leaf tissue allowed the early and accurate identification of waxy genotypes (Carmo et al., 2019). The NIRS technique allows capturing differences in the chemical constitution of plants because of the expression of different genes. Further, leaves are complex assemblies of organic compounds and may be expected to exhibit different spectral responses. NIRS can be successfully used for the characterization of chemical components, like nitrogen, in different plant tissues (Li et al., 2022). In addition to leaf tissue, starch samples have been used to identify the waxy genotype based on NIR spectra in species such as wheat (Lavine et al., 2014; Delwiche and Graybosch, 2016; Delwiche et al., 2018).

The early analysis of greenhouse waxy cassava clones using NIR spectra in leaf tissues, before field planting, allows the exclusive selection of desired genotypes with a high probability to plant the waxy phenotype. Thus, a breeder can avoid planting large populations that do not contain the desired trait (~75% of individuals). However, the use of dried and macerated cassava leaves as sample material requires additional time and resources for the selection process, as it is necessary to sow seeds and grow plants in a greenhouse until the collection time of leaf tissues. The results of the present study indicate that it is possible to classify cassava seeds according to the type of starch with an accuracy close to 1 through classification models based on seed spectral data. Among the two evaluated NIRSs equipment, the NIRFlex N-500 proved to be more accurate, with Kappa values close to 0.80, compared to the portable NIR SCiO. This was possible as each device has different wavelength amplitudes, 740−1070 nm for SCiO and 800−2500 nm for NIRFlex N-500, in addition to the different sample sizes.

Although the NIRFlex N-500 has a higher cost, there is a better resolution in obtaining spectra that maximizes the chance of association with the phenotype of interest (Beć et al., 2022). Due to its numerous advantages, NIR spectra of 800−2500 nm have been used to predict several chemical components in plant seeds (Ferreira et al., 2013). Alternatively, although the SCiO equipment provided high classification accuracy (0.87−0.89), the Kappa indices were very low.

The accuracy values indicate that, in both NIRS equipment, there was a high proportion of correctly classified events in relation to the total number of samples. Accuracy is one of the most intuitive and widely used performance metrics for classification. The Kappa index is a widely used metric to measure classification performance, considering the probability of obtaining the classification by chance. Some authors warn that Kappa may be an inadequate estimate when an unbalanced distribution of classes is involved, where the marginal probability of a class is much (more or less) greater than the others (Donker et al., 1993; Forbes, 1995; Andrés and Marzo, 2004; Delgado and Tibau, 2019). In fact, the dataset evaluated by SCiO showed a greater imbalance between classes compared to the samples evaluated by the NIRFlex N-500.

Portable and smaller equipment, such as the SCiO, has a growing popularity in the agri-food industry. The NIR SCiO is a cost-effective device that stores data in a “cloud”, and it is affordable because it uses an LED light source and a simple 12-element Si photodiode detector, with a configuration matrix of 4 × 3, combined with optical filters on each pixel to form a 12-channel spectrometer (Beć et al., 2022). However, these characteristics give it lower optical performance due to the low number of wavelengths compared to benchtop equipment, such as the NIRFlex N-500 (Beć et al., 2022). Despite these limitations, the spectral region covered is sufficient for the prediction of important parameters related to food quality, such as total soluble solids, maturity, identification of fruits with a high concentration of dry matter (Li et al., 2018a), and sugar content and firmness in tomatoes (Goisser et al., 2018). Additionally, this equipment makes it possible to classify cultivars of barley, chickpeas, and sorghum seeds with 86−96% accuracy (Kosmowski and Worku, 2018).

The accuracies of cross-validation in training set and from confusion matrix in the testing set were high among the classification models analyzed, with emphasis on the XGB algorithm (>0.92). A recent study demonstrated the effectiveness of XGB in analyses with spectral data in food quality control (Li et al., 2018b), in comparison with the Back Propagation Neural Network and Support Vector Regression models, often used in analysis of products of vegetal origin. In addition to the high classification accuracy of waxy clones, the Kappa values obtained by this algorithm were high, at 0.69 and 0.82, respectively. Probably, because it is an extension of random forest and uses a regularization parameter to reduce overfitting, XGB was the algorithm with the highest detection power, allowing it to correctly classify the two classes analyzed (Luckner et al., 2017).

Due to the high number of variables (wavelengths) gathered, mainly by the NIRFlex N-500, the selection of variables makes it possible to remove noise, or highly correlated and non-informative variables, to improve computational performance. Therefore, the classification models were evaluated after selecting the most important spectra based on the XGB algorithm. Following this procedure, a slight increase in Kappa values was observed, and similar classification accuracies was revealed for the different models compared to the analyses performed with all spectra. Therefore, the selection of variables proved to be advantageous for increasing the power of the models to classify waxy cassava clones and in reducing the computational time for processing the analyses.

### 4.2 Prospects for the use of NIRS for early selection in cassava

NIR spectrometry has demonstrated a high potential in predicting key traits such as carotenoids, starch, and dry matter content in cassava (Ikeogu et al., 2017; Bantadjan et al., 2020; Maraphum et al., 2022). The correlation coefficient of prediction was 0.83 for starch content (Bantadjan et al., 2020), 0.88 for carotenoids, and 0.80 for dry matter content (Ikeogu et al., 2017), which ensures a sufficient predictive accuracy of new phenotypes to be generated and evaluated by the cassava breeding programs.

Furthermore, as it is a non-destructive technique, it can be incorporated as a new tool for cassava breeders, improving phenotyping efficiency. When compared to the conventional laboratory techniques for dry matter and carotenoid content in cassava breeding, the NIRS technique is rapid and cost-effective (Ikeogu et al., 2017). The current phenotyping techniques for key traits are laborious and time-consuming for large-scale screenings. Additionally, estimates could be influenced by sample preparation, including weight and number of roots used in the prevalent specific gravity method (Fukuda et al., 2010). For carotenoid quantification using color, the intensity could be subjective and inefficient in an advanced population of biofortified genetic materials (Sánchez et al., 2006). Moreover, laboratory processes using high-performance liquid chromatography (HPLC) or a UV-Visible spectrophotometer are low-throughput, processing less than 10 or 40 samples per day, respectively (Sánchez et al., 2014).

These results bring advances and new techniques for early identification of cassava genotypes with waxy starch at the seed stage, through non-destructive techniques. This allows cassava breeders to generate large F_2_ segregating populations with thousands of individuals. From these populations, it is possible to select desirable genotypes with high classification accuracy before planting in the field.

Despite the initial investment to purchase the NIRS equipment, the economic return is readily apparent in the next seedling trials. After screening the seeds *via* NIRS, it is possible to reduce the planting area of seedlings by up to 75%. In terms of resource allocation, an estimated cost with phenotyping of a field plot, with a seedling per plot, in one environment is 2.20 U.S. dollars. This value was assumed for a single-plant field plot, including phenotyping with the iodine test. On average, 8000 seeds are obtained from segregating populations for waxy starch per year. Screening represents an average savings of $2.20 x 6000 = $13,200.00/year.

## 5 Conclusions

NIR spectroscopy in combination with the eXtreme Gradient Boosting algorithm (XGB) can be used to classify cassava seeds according to the type of waxy and non-waxy starch and select early genotypes with the desired phenotype. The methodology using NIRS techniques showed great potential for applicability, being a fast and efficient tool for the identification of waxy genotypes for practical use as an alternative to utilizing molecular markers in cassava breeding programs.

## Data availability statement

The datasets presented in this study can be found in online repositories. The names of the repository/repositories and accession number(s) can be found below: https://figshare.com/, dx.doi.org/10.6084/m9.figshare.21071257.

## Author contributions

MS, LA, and EO designed the experiments. JF Spectral data collection. LA and MS analyzed the spectral dataset. MS, LA, and EO were involved in the research design and improvement of the manuscript. MS, and EO wrote the manuscript. All authors contributed to the article and approved the submitted version.
