# Supplementary figures and images for: Near-infrared spectroscopy for early selection of waxy cassava clones via seed analysis
Source: Front Plant Sci. 2023 Jan 23;14:1089759. doi: 10.3389/fpls.2023.1089759 (PMC9900181; doi:10.3389/fpls.2023.1089759)

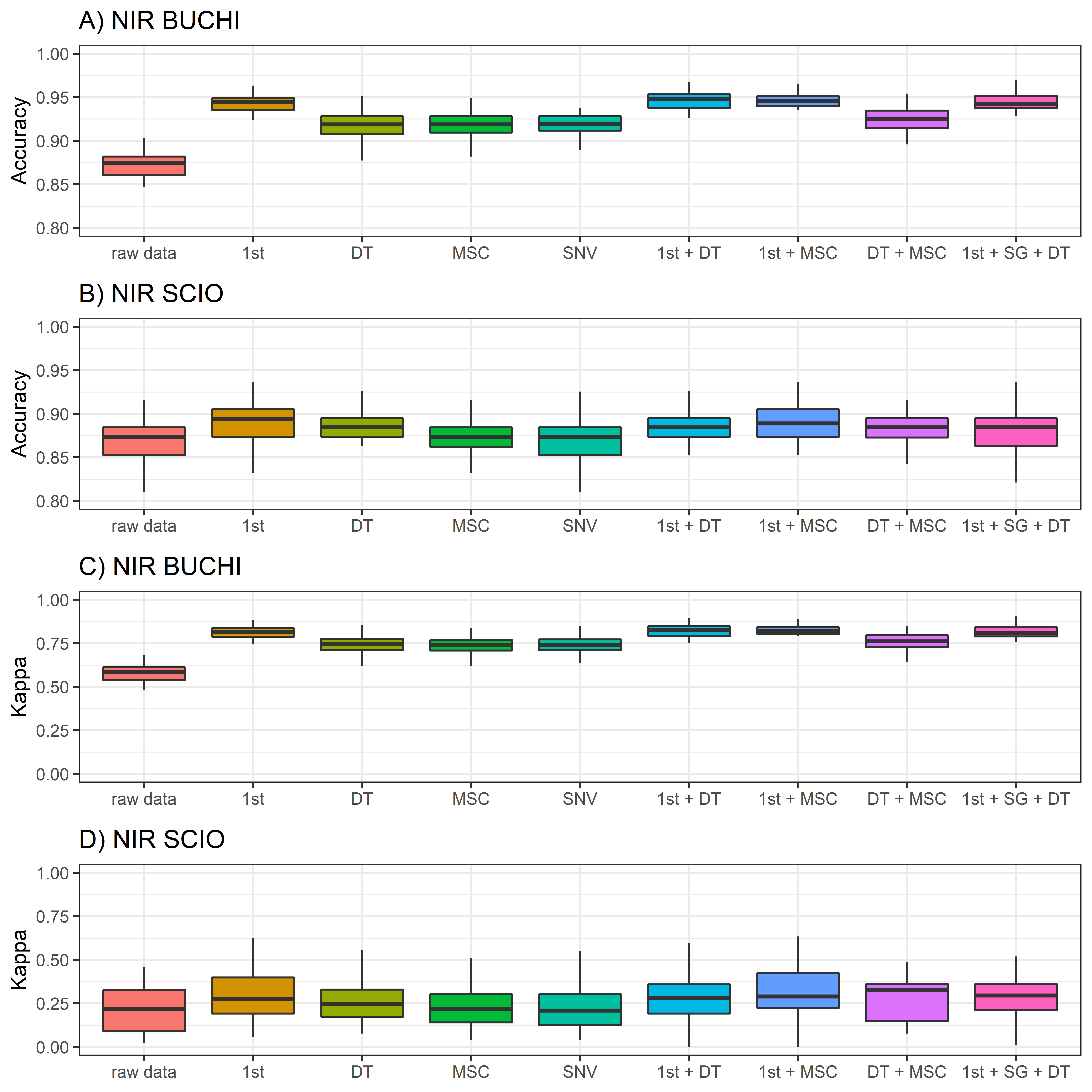

Supplement: Supplementary file 1 [file Image_1.tiff]
