# Supplementary material for: Near-infrared spectroscopy for early selection of waxy cassava clones via seed analysis
Source: Front Plant Sci. 2023 Jan 23;14:1089759. doi: 10.3389/fpls.2023.1089759 (PMC9900181; doi:10.3389/fpls.2023.1089759)
Supplement: Supplementary file 2 [file Table_1.docx]

Supplementary Material

**Table S1**. Segregation analysis of starch type in 85 F_2_ cassava progenies.

| Progeny | Plant/  progeny (n#) | N# Plants observed | | N# Plants expected | | Chi-squared test (3/4; 1/4) | N# Plants expected | Chi-squared test (1/2; 1/2) |
| --- | --- | --- | --- | --- | --- | --- | --- | --- |
|  |  | No waxy | Waxy | No waxy | Waxy |  | No waxy/Waxy |  |
| BR-19F2wx-006 | 5 | 4 | 1 | 3.75 | 1.25 | 0.07 | 2.50 | 1.80 |
| BR-19F2wx-007 | 5 | 4 | 1 | 3.75 | 1.25 | 0.07 | 2.50 | 1.80 |
| BR-19F2wx-012 | 4 | 4 | 0 | 3.00 | 1.00 | 1.33 | 2.00 | 4.00 |
| BR-19F2wx-013 | 8 | 8 | 0 | 6.00 | 2.00 | 2.67 | 4.00 | 8.00 |
| BR-19F2wx-016 | 5 | 5 | 0 | 3.75 | 1.25 | 1.67 | 2.50 | 5.00 |
| BR-19F2wx-023 | 11 | 11 | 0 | 8.25 | 2.75 | 3.67 | 5.50 | 11.00 |
| BR-19F2wx-024 | 4 | 4 | 0 | 3.00 | 1.00 | 1.33 | 2.00 | 4.00 |
| BR-19F2wx-027 | 13 | 13 | 0 | 9.75 | 3.25 | 4.33 | 6.50 | 13.00 |
| BR-19F2wx-029 | 4 | 4 | 0 | 3.00 | 1.00 | 1.33 | 2.00 | 4.00 |
| BR-19F2wx-040 | 4 | 3 | 1 | 3.00 | 1.00 | 0.00 | 2.00 | 1.00 |
| BR-19F2wx-041 | 5 | 3 | 2 | 3.75 | 1.25 | 0.60 | 2.50 | 0.20 |
| BR-19F2wx-045 | 7 | 4 | 3 | 5.25 | 1.75 | 1.19 | 3.50 | 0.14 |
| BR-19F2wx-059 | 10 | 10 | 0 | 7.50 | 2.50 | 3.33 | 5.00 | 10.00 |
| BR-19F2wx-062 | 19 | 19 | 0 | 14.25 | 4.75 | 6.33 | 9.50 | 19.00 |
| BR-19F2wx-067 | 17 | 12 | 5 | 12.75 | 4.25 | 0.18 | 8.50 | 2.88 |
| BR-19F2wx-068 | 6 | 5 | 1 | 4.50 | 1.50 | 0.22 | 3.00 | 2.67 |
| BR-19F2wx-069 | 4 | 2 | 2 | 3.00 | 1.00 | 1.33 | 2.00 | 0.00 |
| BR-19F2wx-075 | 9 | 6 | 3 | 6.75 | 2.25 | 0.33 | 4.50 | 1.00 |
| BR-19F2wx-078 | 8 | 7 | 1 | 6.00 | 2.00 | 0.67 | 4.00 | 4.50 |
| BR-19F2wx-081 | 13 | 9 | 4 | 9.75 | 3.25 | 0.23 | 6.50 | 1.92 |
| BR-19F2wx-085 | 10 | 7 | 3 | 7.50 | 2.50 | 0.13 | 5.00 | 1.60 |
| BR-19F2wx-088 | 7 | 7 | 0 | 5.25 | 1.75 | 2.33 | 3.50 | 7.00 |
| BR-19F2wx-093 | 6 | 6 | 0 | 4.50 | 1.50 | 2.00 | 3.00 | 6.00 |
| BR-19F2wx-094 | 4 | 3 | 1 | 3.00 | 1.00 | 0.00 | 2.00 | 1.00 |
| BR-19F2wx-110 | 5 | 5 | 0 | 3.75 | 1.25 | 1.67 | 2.50 | 5.00 |
| BR-19F2wx-113 | 14 | 14 | 0 | 10.50 | 3.50 | 4.67 | 7.00 | 14.00 |
| BR-19F2wx-114 | 6 | 6 | 0 | 4.50 | 1.50 | 2.00 | 3.00 | 6.00 |
| BR-19F2wx-115 | 4 | 4 | 0 | 3.00 | 1.00 | 1.33 | 2.00 | 4.00 |
| BR-19F2wx-116 | 4 | 4 | 0 | 3.00 | 1.00 | 1.33 | 2.00 | 4.00 |
| BR-19F2wx-119 | 12 | 12 | 0 | 9.00 | 3.00 | 4.00 | 6.00 | 12.00 |
| BR-19F2wx-123 | 6 | 6 | 0 | 4.50 | 1.50 | 2.00 | 3.00 | 6.00 |
| BR-19F2wx-129 | 4 | 4 | 0 | 3.00 | 1.00 | 1.33 | 2.00 | 4.00 |
| BR-19F2wx-130 | 4 | 4 | 0 | 3.00 | 1.00 | 1.33 | 2.00 | 4.00 |
| BR-19F2wx-145 | 6 | 5 | 1 | 4.50 | 1.50 | 0.22 | 3.00 | 2.67 |
| BR-19F2wx-146 | 14 | 13 | 1 | 10.50 | 3.50 | 2.38 | 7.00 | 10.29 |
| BR-19F2wx-149 | 14 | 10 | 4 | 10.50 | 3.50 | 0.10 | 7.00 | 2.57 |
| BR-19F2wx-153 | 28 | 23 | 5 | 21.00 | 7.00 | 0.76 | 14.00 | 11.57 |
| BR-19F2wx-155 | 17 | 13 | 4 | 12.75 | 4.25 | 0.02 | 8.50 | 4.76 |
| BR-19F2wx-156 | 4 | 4 | 0 | 3.00 | 1.00 | 1.33 | 2.00 | 4.00 |
| BR-19F2wx-158 | 7 | 5 | 2 | 5.25 | 1.75 | 0.05 | 3.50 | 1.29 |
| BR-19F2wx-159 | 13 | 10 | 3 | 9.75 | 3.25 | 0.03 | 6.50 | 3.77 |
| BR-19F2wx-164 | 5 | 4 | 1 | 3.75 | 1.25 | 0.07 | 2.50 | 1.80 |
| BR-19F2wx-167 | 10 | 10 | 0 | 7.50 | 2.50 | 3.33 | 5.00 | 10.00 |
| BR-19F2wx-171 | 6 | 4 | 2 | 4.50 | 1.50 | 0.22 | 3.00 | 0.67 |
| BR-19F2wx-200 | 30 | 24 | 6 | 22.50 | 7.50 | 0.40 | 15.00 | 10.80 |
| BR-19F2wx-204 | 5 | 3 | 2 | 3.75 | 1.25 | 0.60 | 2.50 | 0.20 |
| BR-19F2wx-205 | 5 | 4 | 1 | 3.75 | 1.25 | 0.07 | 2.50 | 1.80 |
| BR-19F2wx-218 | 14 | 14 | 0 | 10.50 | 3.50 | 4.67 | 7.00 | 14.00 |
| BR-19F2wx-241 | 16 | 8 | 8 | 12.00 | 4.00 | 5.33 | 8.00 | 0.00 |
| BR-19F2wx-243 | 19 | 14 | 5 | 14.25 | 4.75 | 0.02 | 9.50 | 4.26 |
| BR-19F2wx-246 | 6 | 3 | 3 | 4.50 | 1.50 | 2.00 | 3.00 | 0.00 |
| BR-19F2wx-251 | 8 | 4 | 4 | 6.00 | 2.00 | 2.67 | 4.00 | 0.00 |
| BR-19F2wx-255 | 9 | 5 | 4 | 6.75 | 2.25 | 1.81 | 4.50 | 0.11 |
| BR-19F2wx-256 | 5 | 5 | 0 | 3.75 | 1.25 | 1.67 | 2.50 | 5.00 |
| BR-19F2wx-264 | 7 | 7 | 0 | 5.25 | 1.75 | 2.33 | 3.50 | 7.00 |
| BR-19F2wx-268 | 17 | 12 | 5 | 12.75 | 4.25 | 0.18 | 8.50 | 2.88 |
| BR-19F2wx-274 | 6 | 5 | 1 | 4.50 | 1.50 | 0.22 | 3.00 | 2.67 |
| BR-19F2wx-275 | 5 | 5 | 0 | 3.75 | 1.25 | 1.67 | 2.50 | 5.00 |
| BR-19F2wx-277 | 15 | 11 | 4 | 11.25 | 3.75 | 0.02 | 7.50 | 3.27 |
| BR-19F2wx-284 | 5 | 5 | 0 | 3.75 | 1.25 | 1.67 | 2.50 | 5.00 |
| BR-19F2wx-299 | 5 | 3 | 2 | 3.75 | 1.25 | 0.60 | 2.50 | 0.20 |
| BR-19F2wx-302 | 6 | 6 | 0 | 4.50 | 1.50 | 2.00 | 3.00 | 6.00 |
| BR-19F2wx-329 | 4 | 2 | 2 | 3.00 | 1.00 | 1.33 | 2.00 | 0.00 |
| BR-19F2wx-331 | 5 | 4 | 1 | 3.75 | 1.25 | 0.07 | 2.50 | 1.80 |
| BR-19F2wx-348 | 11 | 8 | 3 | 8.25 | 2.75 | 0.03 | 5.50 | 2.27 |
| BR-19F2wx-359 | 13 | 12 | 1 | 9.75 | 3.25 | 2.08 | 6.50 | 9.31 |
| BR-19F2wx-361 | 13 | 13 | 0 | 9.75 | 3.25 | 4.33 | 6.50 | 13.00 |
| BR-19F2wx-365 | 6 | 6 | 0 | 4.50 | 1.50 | 2.00 | 3.00 | 6.00 |
| BR-19F2wx-371 | 28 | 18 | 10 | 21.00 | 7.00 | 1.71 | 14.00 | 2.29 |
| BR-19F2wx-373 | 4 | 2 | 2 | 3.00 | 1.00 | 1.33 | 2.00 | 0.00 |
| BR-19F2wx-376 | 7 | 2 | 5 | 5.25 | 1.75 | 8.05 | 3.50 | 1.29 |
| BR-19F2wx-378 | 4 | 4 | 0 | 3.00 | 1.00 | 1.33 | 2.00 | 4.00 |
| BR-19F2wx-380 | 5 | 3 | 2 | 3.75 | 1.25 | 0.60 | 2.50 | 0.20 |
| BR-19F2wx-381 | 11 | 7 | 4 | 8.25 | 2.75 | 0.76 | 5.50 | 0.82 |
| BR-19F2wx-383 | 4 | 3 | 1 | 3.00 | 1.00 | 0.00 | 2.00 | 1.00 |
| BR-19F2wx-385 | 85 | 52 | 33 | 63.75 | 21.25 | 8.66 | 42.50 | 4.25 |
| BR-19F2wx-387 | 4 | 4 | 0 | 3.00 | 1.00 | 1.33 | 2.00 | 4.00 |
| BR-19F2wx-404 | 51 | 30 | 21 | 38.25 | 12.75 | 7.12 | 25.50 | 1.59 |
| BR-19F2wx-408 | 10 | 6 | 4 | 7.50 | 2.50 | 1.20 | 5.00 | 0.40 |
| BR-19F2wx-411 | 8 | 7 | 1 | 6.00 | 2.00 | 0.67 | 4.00 | 4.50 |
| BR-19F2wx-413 | 7 | 4 | 3 | 5.25 | 1.75 | 1.19 | 3.50 | 0.14 |
| BR-19F2wx-432 | 8 | 6 | 2 | 6.00 | 2.00 | 0.00 | 4.00 | 2.00 |
| BR-19F2wx-438 | 41 | 41 | 0 | 30.75 | 10.25 | 13.67 | 20.50 | 41.00 |
| BR-19F2wx-440 | 12 | 8 | 4 | 9.00 | 3.00 | 0.44 | 6.00 | 1.33 |
| BR-19F2wx-447 | 24 | 24 | 0 | 18.00 | 6.00 | 8.00 | 12.00 | 24.00 |
| Total | 1127 | 887 | 240 | 845.25 | 281.75 | 8.25 | 563.50 | 373.07 |

*Progenies with 4 or more plants.
